# Proximate Composition and Antioxidant Potential of Leaves from Three Varieties of Mulberry (*Morus* sp.): A Comparative Study

**DOI:** 10.3390/ijms13066651

**Published:** 2012-05-30

**Authors:** Shahid Iqbal, Umer Younas, Sirajuddin, Kim Wei Chan, Raja Adil Sarfraz, Kamal Uddin

**Affiliations:** 1 Department of Chemistry, University of Sargodha, Sargodha 40100, Pakistan; E-Mails: ranashahid313@gmail.com (S.I.); umer0608analyst@gmail.com (U.Y.); 2 National Center of Excellence in Analytical Chemistry, University of Sindh, Jamshoro 76080, Pakistan; E-Mail: drsiraj03@yahoo.com; 3 Laboratory of Molecular Biomedicine, Institute of Bioscience, Universiti Putra Malaysia, 43400 Serdang, Selangor, Malaysia; 4 Department of Chemistry and Biochemistry, University of Agriculture, Faisalabad 38040, Pakistan; E-Mail: rajaadilsarfraz@gmail.com; 5 Department of Crop Science, Faculty of Agriculture, Universiti Putra Malaysia, 43400 Serdang, Selangor, Malaysia; E-Mail: mkuddin07@yahoo.com

**Keywords:** mulberry leaves, varieties, proximate composition, antioxidant activity

## Abstract

In this study, leaves of three indigenous varieties of Mulberry namely, *Morus alba* L., *Morus nigra* L. and *Morus rubra* L. were investigated for their antioxidant potential and their proximate composition was determined. The yields of 80% methanolic extracts ranged between 8.28–13.89%. The contents of total phenolics (TPC), total flavonoids (TFC) and ascorbic acid (AA) ranged between 16.21–24.37 mg gallic acid equivalent (GAE)/g, 26.41–31.28 mg rutin equivalent (RE)/g and 0.97–1.49 mg/g, respectively. The antioxidant activity of leaf extracts was evaluated by measuring 1,1-diphenyl-2-picrylhydrazyl (DPPH^•^) radical scavenging actity, 2,2′-azino-bis-(3-ethylbenzthiazoline-6-sulphonic acid (ABTS^•+^) radical cation scavenging capacity and ferric ion reducing power and values ranged between 1.89–2.12, 6.12–9.89 and 0.56–0.97 mM Trolox equivalent/g of dried leaves, respectively. The investigated features reveal good nutritive and antioxidant attributes of all the varieties with mutually significant differences.

## 1. Introduction

Free radicals and reactive oxygen species (ROS) are regularly and continuously produced as byproducts of normal cellular metabolism in aerobics [1]. Being unstable and reactive, they possess the ability to damage essential biomolecules (lipid, DNA and protein) in the form of tissue injury and cell death; if they exceed a certain limit. Their harmful effects can be avoided by their regular removal from the body. To meet the purpose, there is a built-in natural antioxidative defense system (enzymatic and non-enzymatic) in the human body, which continuously and proportionally neutralizes free radicals by scavenging [2]. Sometimes, the population of free radicals starts increasing due to different exogenous factors and agents [3] that may not be completely neutralized by built-in antioxidant system. This results in an imbalance between the production and depletion of free radicals in the body; this state is termed oxidative stress [4]. Epidemiological studies have proved the role of oxidative stress in generation and propagation of many chronic diseases such as cancer, cardiovascular, Alzheimer’s diseases as well as neurodegenerative disorders [5]. To avoid the hazards associated with oxidative stress, the external aid of antioxidants in the form of food supplements is required by the human body [2]. For this purpose, synthetic antioxidants such as butylated hydroxyanisole (BHA) and butylated hydroxytoluene (BHT) have been in use for several years as food additives, but now their use is restricted in dietary items due to reports published on their involvement in number of chronic diseases including cancers and cardiovascular disorders [6]. These reports prompted the researchers and scientists to explore potent natural sources of antioxidants based on botanical origin including cereal crops, fruits, vegetables, oilseeds and various parts (roots, barks, leaves and fruits) of medicinal plants [7]. As a result, many plant materials have been identified and documented as promising sources of natural antioxidants [8,9]. Besides this, antioxidant attributes of these plant materials have been investigated as a function of growing location, species, and cultivation conditions *etc*., and noticeable differences were observed. Now, there is ample evidence regarding variation in proximate composition, phenolics and antioxidant activity with respect to different species of a plant [5,10]. Following the same trend, the present work was designed to study the differences in leaves from three varieties of an abundantly available indigenous fruity plant, the mulberry.

The mulberry, a perennial woody plant having fast growth and short proliferation period [11], belongs to family Moraceae and genus *Morus* [12]. In general, 10–16 species of genus *Morus* are found in subtropical, warm and temperate regions of Asia, Africa and North America [13]. Some of these are preferred due to foliage yield, delicious fruit, while few have ornamental importance and others are used due to their strong environmental adaptability [11]. Commonly, three species of *Morus*, *Morus alba*, *Morus nigra* and *Morus rubra*, are grown [14] in colder regions of Pakistan e.g., Azad Kashmir, Chitral and Quetta [15].

Roots, bark and leaves of *Morus alba* L. are used for various health benefits and ailments in Chinese traditional medicines [16]. The antioxidant activity of these parts of *M. alba* in different model systems is also documented [17]. Due to the presence of precious phytochemicals (coumarins, flavonoids, phenols), the leaves of *Morus alba* L. possess pharmacological importance and have been reported to reduce blood pressure and cholesterol level [18]. *Morus nigra* L. is a wildly growing rustic plant, also grown in gardens and is used as sericulture [13]. It is also used as part of Chinese folk medicine, for the treatment of arthritis, diabetes and rheumatis. Recently two new flavonoids have been isolated from its leaves [19] and the antioxidant profile of different parts of this specie has also been studied [20]. *Morus rubra* L. is a medium sized (15–20 m) tree; its leaves are of great importance in folk medicine and are reported to have many phytochemical constituents and biological activities [21].

Recently, many studies have shown antioxidant, antiviral, anti-inflammatory, hypolipidemic, anti-hyperglycemic, neuroprotective [11], anti-HIV, anti-hypotensive and cytotoxic activities of different species of *Morus* [22]. Mulberry leaves have shown strong antioxidant properties in rice bran oil and inhibition of oxidative deterioration of oil was observed to be even better than synthetic antioxidants [23]. Some reports have attributed these salient features to the many phytochemical constituents present in mulberry leaves [24]. These leaves have been being consumed in Korea and Japan for diabetes mellitus patients [25] and are also used in noodles, cakes and tea as nutraceutical supplements [26].

In the light of these considerations in the literature, it is clear that a very little information about the antioxidant activity of leaves from different mulberry species is available. Hence a work was planned to report comprehensive information about antioxidant components and scavenging potential of leaves from commonly available species of Mulberry (*Morus* sp.) employing multiple assays based on different theoretical principle. Furthermore, on the basis of conducted investigations, a comparison was made among leaves of selected species regarding their antioxidant activity and nutraceutical potential.

## 2. Results and Discussion

### 2.1. Proximate Composition

The mean values of proximate composition for the leaves of three different varieties of mulberry are summarized in Table 1 and the varieties were found to be statistically different (*P* < 0.05) in the context of ash, moisture, lipid, fiber and protein contents. The minimum and maximum values for ash content were observed in *M. alba* and *M. rubra* respectively, while moisture contents ranged from 5.3 ± 0.2 for *M. alba* to 6.7 ± 0.3 for *M. nigra*. The values of ash are much higher while those for moisture are lower in comparison to the values reported previously for vegetables [27]. High ash content indicates the presence of heavy amounts of inorganic nutrients in plant material [28], whereas low moisture content may contribute towards roughness of leaves.

Estimation of lipids is considered amongst the key factors for nutritional evaluation of any material [29]. The varieties of mulberry exhibited significant variation in the amount of lipids, *M. alba* contained the highest percentage of lipids followed by *M. nigra* and *M. rubra* respectively, but these values were lower than those reported for Nigerian plants [30]. However, the presence of an appreciable content of lipids demonstrates the potential of these leaves to have dietary purposes with promising nutritional attributes.

Dietary fibers are non-starch polysaccharides (anti-nutrient), which bind minerals and accelerate their passage through digestive tract, as a result bioavailability and absorption of nutrients is reduced. This whole process becomes more effective when fibers collaborate with other food constituents like phytate, tannin or oxalate [31]. *Morus nigra* contains high amount of fiber compared to the other two species, *Morus alba* and *Morus rubra*. The investigated species were found to be statistically different (*p* < 0.05) and richer in fiber contents as compared to leafy vegetables [27].

The trend of protein content in leaves from all the investigated varieties was observed in the following order, *M. rubra* > *M. nigra* > *M. alba*, with significant differences among them (*p* < 0.05). Being a better source of protein as compared to other leafy vegetables [32], these leaves may be explored as good source of comparable and promising antioxidant activity [33].

### 2.2. Extract Yield and Total Phenolic Content (TPC)

**Figure 1:**
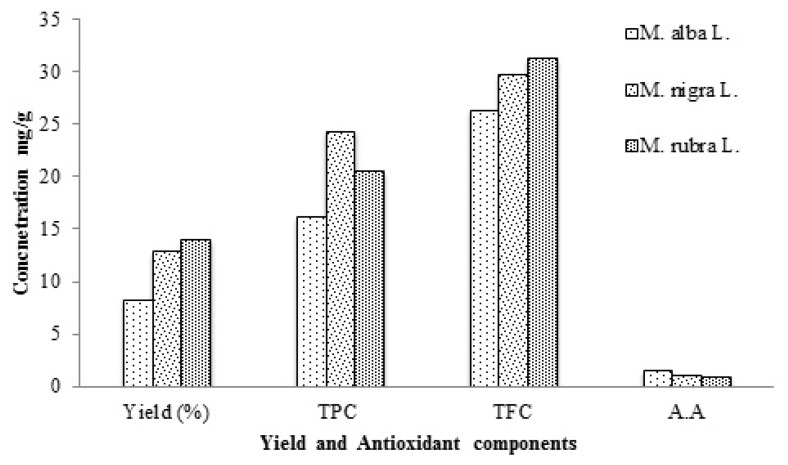
Yield and contents of Phenolics, Flavonoids and Ascorbic acid in extracts from dried leaves of three mulberry varieties (*p* < 0.05).

Epidemiological studies have confirmed the disease preventive role and antioxidant activities of phenolics and many reports have highlighted variation in phenolic compounds as function of plant species [34]. The content of total phenolics was estimated as mg GAE/g of dried leaves and ranged from 16.21 ± 1.34 for *M. alba* to 24.37 ± 2.14 for *M. nigra* (Figure 1). Significant differences (*p* = 0.004) were noted for TPC among the leaves of three mulberry varieties. These results are much higher than reported for oil palm leaves [35], and Iranian medicinal plants [36].

### 2.3. Total Flavonoid Content (TFC)

Flavonoids, the largest subgroup of plant phenolics, constitute almost half of the reported phenolic compounds [37]. Many biological effects including free radical scavenging activity have been reported for flavonoids, which are generally attributed to their structural features [6]. The flavonoid content in mulberry leaves was estimated as mg rutin equivalent/g of dried leaf samples, ranging from 26.41 ± 1.14 to 31.28 ± 2.12 for *M. alba* and *M. rubra* respectively (*p* < 0.05). The amount of flavonoids in mulberry leaves was found to be greater than Algerian medicinal plants [38].

### 2.4. Ascorbic Acid Content (AA)

Ascorbic acid (vitamin C) is water soluble, non-enzymetic natural antioxidant [39], which is widely used as an alternative to synthetic antioxidants [40]. Significant differences were observed regarding ascorbic acid (AA) content estimated in leaves of different mulberry varieties. The maximum amount of AA was found in the leaves of *M. alba* followed by *M. nigra* while the minimum was found for *M. rubra*.

### 2.5. DPPH Radical Scavenging Activity

**Figure 2:**
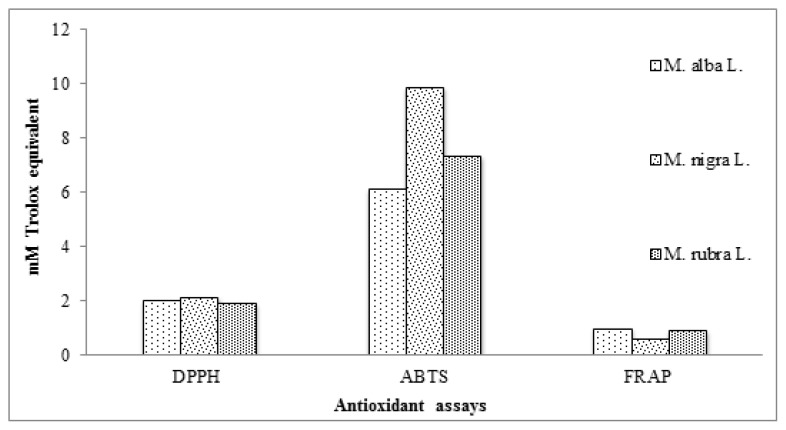
Antioxidant activity of dried mulberry leaves through 1,1-diphenyl-2-picrylhydrazyl (DPPH^•^) scavenging activity, 2,2′-azino-bis-(3-ethylbenzthiazoline-6-sulphonic acid (ABTS^•+^) scavenging activity and ferric ion reducing antioxidant power (FRAP) assays.

DPPH assay is widely used for the evaluation of antioxidant activity of biological samples. The working principle of this assay is based on discoloration of DPPH free radical upon reacting with hydrogen donating species *i.e.*, antioxidants present in plant extracts [41]. The results of DPPH assay for the leave extracts from three varieties of mulberry were calculated as mM Trolox equivalent (TE)/g of dried leaves (Figure 2). *M. nigra* exhibited the highest radical scavenging potential followed by *M. alba* and *M. rubra* respectively; differences being non-significant (*p* > 0.05) among the varieties. However, the values of DPPH radical scavenging, calculated as millimole per liter TE/g for the tested samples were found to be much higher than *Potentilla fulgens* [42].

### 2.6. ABTS Radical Cation Scavenging Activity

The working mechanism of the ABTS method for the evaluation of antioxidant activity is the same as that of the DPPH method, but the ABTS method is more reliable than the DPPH method, due to solubility of the ABTS reagent in both aqueous and organic solvents and rapid reaction with lipophilic as well as hydrophilic antioxidant species as compared to DPPH [43]. The leaves from three varieties of mulberry showed significantly different (*p* < 0.05) ability to mutually scavenge the ABTS radical cation, ranging from 6.12 ± 0.53 to 9.89 ± 0.87 mM Trolox equivalent for *M. alba* and *M. nigra*, respectively (Figure 2). ABTS radical cation scavenging values of mulberry leaves are higher than those reported in recent literature [42,44] and the Spanish Mediterranean diet [45].

### 2.7. Ferric Ion Reducing Antioxidant Power (FRAP)

FRAP assay measures the antioxidant capacity in terms of extract’s ability to reduce ferric (Fe^3+^) to ferrous (Fe^2+^) state. This reduction process is based on single electron transfer mechanism and works in both water and alcoholic systems [46]. The methanolic extracts of dried leaves from three mulberry varieties were subjected to antioxidant activity evaluation using FRAP assay and results were calculated as millimole per liter Trolox equivalent/g of dried leaf samples and significant differences (*p* < 0.05) were found regarding their ferric ion reducing capacity. Among all the tested varieties, *M. alba* leaves were found to have high FRAP value while least FRAP values were observed for *M. nigra*. Ferric reducing antioxidant power of mulberry leaves is comparatively higher than many other reported species [44,45].

Trolox equivalent (TE) values calculated for ABTS radical cation scavenging assay were three to four fold higher than those calculated for DPPH radical scavenging assay, while TE values for both of these assays are noticeably higher than those calculated for FRAP assay. This difference in TE values for different assays, using different radicals, has also been reported for barley varieties and may be attributed to different kinetics of reactions between radicals employed and phenolic constituents present in the sample or may be due to different response of phenolics towards different type of radicals [47]. These findings verify the efficiency of mulberry leaves extracts against the ABTS radical cation, DPPH radical as well as in FRAP assay.

### 2.8. Correlation among Antioxidant Components and Assays

The Pearson correlation among the results of chemical composition, antioxidant constituents and antioxidant potential of leaves from three varieties of mulberry was assessed (Table 2). Negative correlation of lipid content with all other parameters was observed except ascorbic acid (*r* = 0.996) and DPPH (*r* = 0.307). Highly positive correlation between lipid content and ascorbic acid suggests that the specie with higher lipid content would also be rich in ascorbic acid. Fiber showed high correlation with DPPH radical (*r* = 0.999) and mild correlation with ABTS radical cation (*r* = 0.690) and TPC (*r* = 0.505). This reveals that the species having higher fiber content may exhibit higher scavenging activity against DPPH^•^, while moderate against ABTS radical cation. The “*r*” value for protein and flavonoid was high (*r* = 0.866), revealing good correlation among these two components in leaves from different species of mulberry.

The correlation of ascorbic acid (AA) with DPPH (*r* = 0.227) and FRAP (*r* = 0.392) was found to be very weak, while it was moderately negative (*r* = −0.515) with ABTS. High correlation of TPC with ABTS (*r* = 0.973) confirms the role of phenolics, present in leaves from different mulberry species, for the scavenging of ABTS radical cations. On the other hand, TPC had an intermediate correlation (*r* = 0.537) with DPPH and a strongly negative correlation (*r* = −0.932) with FRAP. A similar correlation was observed among TFC and antioxidant assays, *i.e.*, the correlation of TFC with all the assays was negative except ABTS which was moderate (*r* = 0.505), it confirms the contribution of many other components such as vitamins, anthocyanins and carotenoids towards antioxidant activity exhibited by the sample extracts [48]. Strong correlation of DPPH with ABTS, with value 0.717, confirms the authenticity of results of antioxidant potential for the leave extracts of different mulberry varieties. In contrast, the contradiction of FRAP assay appeared to have a negative correlation with DPPH and ABTS indicating that no single antioxidant assay is sufficiently reliable to evaluate the antioxidant activity of plant extracts prepared from various varieties [49,50] as different components exhibit their specific activity under different assays, each working on different principles. Furthermore, for comparison, antioxidant property of different extracts may not only be attributed to contents of ascorbic acid, phenolics and flavonoids rather the involvement of many other phytochemicals is also taken into account by many authors [51].

## 3. Experimental Section

### 3.1. Chemicals and Reagents

Analytical grade reagents were used throughout this work; methanol was purchased from Fisher Scientific, Leicestershire, UK; Folin–Ciocalteu reagent, 1,1-Diphenyl-2-picrylhydrazyl (DPPH) radical, 2,2′-azinobis(3-ethylbenzothiazoline-6-sulphonic acid) diammonium salt (ABTS), 2,4,6-tripyridyl-*S*-triazine (TPTZ), metaphosphoric acid, 2,6-dichloroindophenol and rutin were procured from Sigma/Aldrich; gallic acid, aluminium chloride, glacial acetic acid and Sodium nitrite were obtained from BDH; while phosphate buffer from R&M Chemicals, Bristol, UK.

### 3.2. Collection of Leaf Samples

Fresh leaf samples of the selected mulberry species (*Morus alba*, *Morus nigra* and *Morus rubra*) were collected from hilly areas of Azad Kashmir and Chitral, Pakistan. After collection, the samples were washed with running tap water followed by rinsing with deionized water and chopping into small pieces. All the three types of chopped samples were placed in an electric oven at 40 °C for two days until a constant weight was attained. The dried samples were ground to powder form to pass through a 1 mm sieve and were stored in sealed polyethylene bags at ambient conditions until further analyses.

### 3.3. Proximate Composition

The proximate composition (ash, moisture, lipid, fiber and protein) of fresh leaf samples was determined using AOAC protocol (1995) [52]. Briefly, ash content was determined by dry ashing method *i.e.*, placing the sample in furnace. Lipids were extracted from powdered samples in soxhlet extractor. The extract obtained was left for overnight drying at 80 °C and contents of lipids were determined gravimetrically. Fiber content was determined by titrimetric method of analysis. Kjeldhal apparatus was used for the estimation of nitrogen content and protein content was calculated as *N* × 6.25.

### 3.4. Extraction of Antioxidants

Five grams of powdered leaf sample of each variety were individually added to 50 mL methanol (80%) and extraction was carried out by sonicating the mixture in an ultrasonic bath for 45 min at ambient temperature [53]. After filtration through 0.45 mm nylon membrane filter, the extracts were dried under reduced pressure at 45 °C using rotary evaporator and were stored at −20 °C prior to further analyses [54].

### 3.5. Estimation of Total Phenolic Content (TPC)

The TPC in methanolic extracts from leaves of three *Morus* varieties were determined using Folin–Ciocalteu (FC) reagent based spectrophotometric assay [54]. 200 μL extract was mixed with 100 μL of freshly prepared and diluted (1:10) FC reagent followed by addition of 7.5% solution of sodium carbonate (2 mL). The total volume of reaction mixture was increased to 7 mL by adding deionized water and allowing the reaction to complete by placing the mixture in dark for 2 h at ambient conditions. Absorbance of resulting mixture was measured at 765 nm spectrophotometrically and amount of phenolics was calculated as gallic acid equivalents (mg/g), using standard curve of gallic acid. Experiments were conducted thrice for each extract and results were averaged.

### 3.6. Estimation of Total Flavonoid Content (TFC)

For determination of TFC, a previously reported method was followed [54]. Deionized water (4 mL) was taken in volumetric flasks of 10 mL capacity and 4 mL deionized water was added in each flask followed by the addition of leaves extracts (1 mL) of each variety and 0.3 mL of 5% NaNO_2_. After 5 min, 0.3 mL of 10% AlCl_3_ was added and at 6th min, 2 mL of 1 M NaOH were added. Deionized water (2.4 mL) was added for dilution purposes and all the contents were carefully mixed. Absorbance of resulting mixture was recorded at 510 nm. Flavonoid content of the samples was calculated as rutin equivalents (mg/100 g) from standard curve of rutin. All the experiments were conducted thrice and results were averaged.

### 3.7. Quantification of Ascorbic Acid

Ascorbic acid content in methanolic extracts of leave samples was estimated using a titrimetric method [55]. Sample extract (0.3 mL) was added to titration flask already containing 20 mL mixture of glacial acetic acid (3.0%) and metaphosphoric acid (8.0%). The mixture was then titrated against 2,6-dichloroindophenol solution (0.025%) until pink color of solution sustained for 10 s. The ascorbic acid content was calculated on the basis of standard curve and was expressed as mg ascorbic acid/g of powdered leaves. The results of three replicate analyses were averaged.

### 3.8. DPPH Radical Scavenging Assay

Free radical scavenging activity was measured following a previously reported method [44]. The stock solution was prepared by dissolving 2.4 mg of DPPH radical/mL of methanol, which was further diluted to adjust the absorbance at 0.7 at a wavelength of 515 nm. Methanolic extracts of leaves (5–25 μL) were mixed with 975–995 μL of DPPH radical solution. The reaction mixture was incubated for 30 min at room temperature followed by measurement of absorbance at 515 nm. Trolox was used as calibration standard and DPPH radical scavenging antioxidant capacity was calculated as millimole per liter Trolox equivalent (mM TE) dry weight of leave samples as average of three concordant readings.

### 3.9. ABTS Radical Cation Scavenging Assay

ABTS radical cation scavenging assay was carried out following an earlier method [56]. ABTS (5 mM) radicals were generated in an aqueous medium by oxidizing ABTS with MnO_2_ for half an hour. The leave extracts were diluted in 5 mM phosphate buffered saline (PBS, pH 7.4), until the absorbance sustained up to 0.700 (±0.020) at 734 nm. Then the extracts treated with buffer (2.5 mL) were added to 7 mL ABTS**^•^**^+^ solution and absorbance of mixture was measured after 10 min of initial mixing at room temperature using PBS as blank. The antioxidant activity of leave extracts in terms of ABTS radical cation scavenging potential was determined using Trolox as standard and results were averaged as mM TE dry weight of leaves samples.

### 3.10. Ferric Reducing Antioxidant Power (FRAP) Assay

Ferric reducing antioxidant power (FRAP) assay was performed following a reported method [57] with slight modifications. Stock solutions of acetate buffer (300 mM), FeCl_3_·6H_2_O (20 mM) and TPTZ in 40 mM in HCl (10 mM), were prepared. Twenty five milliliter of acetate buffer, 2.5 mL TPTZ solution and 2.5 mL FeCl_3_·6H_2_O were mixed to prepare working solution followed by heating to 37 °C and maintaining its pH at 3.6. Fifty microliters of individual sample were mixed with 2 mL of working solution, and absorbance was recorded at 593 nm against blank for 4 min. The average results of FRAP assay were expressed as millimole per liter Trolox equivalents in accordance to standard curve of Trolox.

### 3.11. Statistical Analysis

The proximate composition of leaves from three varieties of mulberry is presented as mean ± standard deviation in Table 1. Analysis of variance (ANOVA) of all the estimated variables was carried out separately and differences were considered significant at *p* < 0.05. Pearson correlation was conducted to evaluate the association among different estimated parameters (Table 2). All the statistical analyses were conducted using Microsoft office Excel 2007 for windows.

## 4. Conclusions

Leaves from different species of *Morus* were found to be significantly different in context of all the investigated parameters, revealing the fact that proximate composition, phenolics, flavonoids and antioxidant activities are considerably affected with variety of plant chosen. Due to presence of less anti-nutrient species like fiber while high protein and ash contents, *M. rubra* can be investigated for its nutraceutical applications. On the other hand, a high amount of phenolics, DPPH radical and ABTS radical cations scavenging potential suggest the superiority of *M. nigra* over the other species regarding their disease preventive potential.

## Figures and Tables

**Table 1:** Proximate composition of leaves from three varieties of mulberry.

	*Morus alba* L.	*Morus nigra* L.	*Morus rubra* L.	*p* value
Ash	8.91 ± 0.51	9.12 ± 0.41	11.73 ± 1.09	0.005
Moisture	5.3 ± 0.2	6.7 ± 0.3	4.5 ± 0.2	<<0.05
Lipid	6.57 ± 0.23	5.13 ± 0.19	4.24 ± 0.11	<<0.05
Fiber	10.11 ± 0.37	12.32 ± 1.18	8.17 ± 0.89	0.003
Protein	18.41 ± 1.36	19.76 ± 2.12	24.63 ± 0.86	0.006

**Table 2:** Pearson correlation among chemical/antioxidant constituents and scavenging activities for leaves from three varieties of mulberry.

	Lipid	Fiber	Protein	TPC	TFC	AA	DPPH	ABTS	FRAP
Lipid	1								
Fiber	0.343	1							
Protein	−0.899	−0.714	1						
TPC	−0.636	0.505	0.236	1					
TFC	−0.997	−0.275	0.866	0.690	1				
AA	0.996	0.264	−0.860	−0.698	−0.999	1			
DPPH	0.307	0.999	−0.691	0.537	−0.235	0.227	1		
ABTS	−0.442	0.690	0.006	0.973	0.505	−0.515	0.717	1	
FRAP	0.315	−0.783	0.130	−0.932	−0.381	0.392	−0.805	−0.990	1
